# A Remote Oral Self-Care Behaviors Assessment System in Vulnerable Populations: Usability and Feasibility Study

**DOI:** 10.2196/54999

**Published:** 2024-08-02

**Authors:** Danielle LaVine, Zara Greer, Jiyun Kim, Santosh Kumar, Thomas Belin, Vivek Shetty

**Affiliations:** 1 Department of Biostatistics Fielding School of Public Health University of California, Los Angeles Los Angeles, CA United States; 2 Section of Oral & Maxillofacial Surgery School of Dentistry University of California, Los Angeles Los Angeles, CA United States; 3 Department of Computer Science University of Memphis Memphis, TN United States

**Keywords:** dental disease, underserved populations, mHealth, usability testing, feasibility testing, mobile phone

## Abstract

**Background:**

Preventative self-care can reduce dental disease that disproportionately burdens vulnerable populations. Personalized digital oral self-care behavioral interventions offer a promising solution. However, the success of these digital interventions depends on toothbrushing data collection e-platforms attuned to the needs and preferences of vulnerable communities.

**Objective:**

The aim of this study is to assess the usability and feasibility of the Remote Oral Behaviors Assessment System (ROBAS), which has been adapted to address the unique requirements of socioeconomically disadvantaged minority individuals.

**Methods:**

A cohort of 53 community-clinic participants, including 31 (58%) Latino and 22 (42%) Black individuals with no prior experience using electric toothbrushes, were recruited to use ROBAS, with planned assessments at baseline, 2 months, and 4 months. Beyond evaluating ROBAS’s technical performance, extensive feedback was gathered to gauge users’ experiences, viewpoints, and overall contentment. The System Usability Scale (SUS) served as a primary metric for assessing user satisfaction and acceptability.

**Results:**

ROBAS exhibited largely reliable and consistent data-gathering capabilities. SUS scores (mean 75.6, SD 14.5) reflected participant contentment within a range of values for other commonly used digital devices and technologies. Among participants who answered questions about willingness to pay for ROBAS, 97% (30/31) indicated that they were willing to pay for ROBAS either as a one-time payment or as a subscription-based service. Additionally, 87.5% of participants expressed that they would endorse it to acquaintances. Most participants expressed no reservations about privacy; among those who expressed privacy concerns (n=20, 50%), the concerns included exposure of information (n=18, 45%), monitoring of brushing habits (n=12, 30%), and collection of information (n=14, 35%), although these concerns did not significantly correlate with specific participant traits. In qualitative terms, users valued ROBAS's ability to monitor brushing habits but called for refinements, especially in Wi-Fi and application connectivity. Recommendations for system improvements encompassed enhanced app functionality, individualized coaching, more comprehensive brushing data, and the addition of flossing activity tracking.

**Conclusions:**

The research highlights ROBAS's promise as a digital platform for unobtrusively tracking daily oral self-care activities in marginalized communities. The system proved to be both feasible, as evidenced by its stable and accurate data capture of brushing behaviors, and user-friendly, as reflected by strong SUS scores and positive user feedback. Influential factors for its uptake included ease of learning and operation, and the feedback provided.

## Introduction

When health care resources are scarce and access to care is constrained, emphasizing preventative self-care behaviors can have a greater impact than treating existing diseases [1,2]. This is particularly true for dental diseases, which are largely preventable and often result from inadequate oral self-care. Substantial evidence suggests that regular and systematic toothbrushing prevents dental plaque accumulation that leads to gum disease, tooth decay, and tooth loss [3-5]. However, good oral health relies on the ability and willingness of individuals to practice preventive oral self-care behaviors (OSCBs) at home. Reducing the risk of dental disease can significantly improve one’s quality of life while decreasing demand for dental care services and resources.

To encourage and reinforce OSCBs, digital behavioral interventions that engage individuals in managing their oral health are being developed [6]. These digital oral self-care interventions (DOSCIs) are particularly relevant to socioeconomically marginalized populations who often face challenges in accessing affordable dental care or essential dental services. By using innovative technologies and digital platforms, self-care interventions can empower these communities with valuable knowledge and practical guidance on ideal pre-emptive care [7-13]. Moreover, DOSCIs can facilitate crucial pre-emptive care during extended disruptions to care-delivery systems, as was the case during the COVID-19 pandemic [7,8].

Implementing the DOSCIs requires e-platforms capable of consistently and accurately gathering data on health behaviors, such as oral self-care habits, in everyday settings without being intrusive. In prior work, we outlined the architecture of a versatile digital platform designed for the remote monitoring of toothbrushing practices in real-world settings [9]. Our Remote Oral Behaviors Assessment System (ROBAS) leverages the ubiquity of electric toothbrushes and smartphones, aligning with current sociotechnological trends that favor the use of intelligent, interconnected devices for monitoring health activities and delivering tailored digital guidance and feedback [10-13].

The COVID-19 pandemic prompted us to adapt ROBAS to address access inequalities, particularly for underresourced minority populations. Using an iterative and participatory co-design methodology, we tailored ROBAS to align with the unique preferences and requirements of vulnerable communities. The primary aim of this study was to assess the usability and feasibility of the enhanced ROBAS system, with a specific focus on socioeconomically disadvantaged minorities. Usability metrics provide insight into user satisfaction and ease of use of the system, while feasibility metrics incorporate the technical viability and effective data capture capabilities of ROBAS. Using a research design that blended both quantitative and qualitative approaches, our study sought to assess the real-world applicability of ROBAS, particularly among individuals at higher risk for dental disease and who were new to electric toothbrushes. On the quantitative front, we focused on metrics such as system reliability and user engagement to gauge its feasibility. Meanwhile, the qualitative component of the research involved in-depth interviews and user feedback sessions to explore the system's usability, including ease of use, user satisfaction, and the intuitiveness of its interface.

## Methods

### Revamped ROBAS

In its initial version, ROBAS relied on a dedicated study smartphone to collect OSCB data (ie, frequency and duration of brushing episodes, epochs of excessive pressure) via inertial sensors in an Oral-B 7000 electric toothbrush [9]. For enhanced user-friendliness and broader adoption, the updated ROBAS system simplified the data collection process by doing away with the necessity for a proximate smartphone. A “smart charger” base automatically collects sensor data from the toothbrush via Bluetooth Low Energy technology. These data are then transmitted over local Wi-Fi to a HIPAA (Health Insurance Portability and Accountability Act)-compliant cloud server for real-time monitoring and analytics. An accompanying dashboard created with the Shiny package for R [14] provided the research team with a graphical display of time-series data related to brushing activities, as well as ongoing monitoring of both ROBAS system performance and participant OSCBs in the home setting.

### Automated Feedback

**Figure 1:**
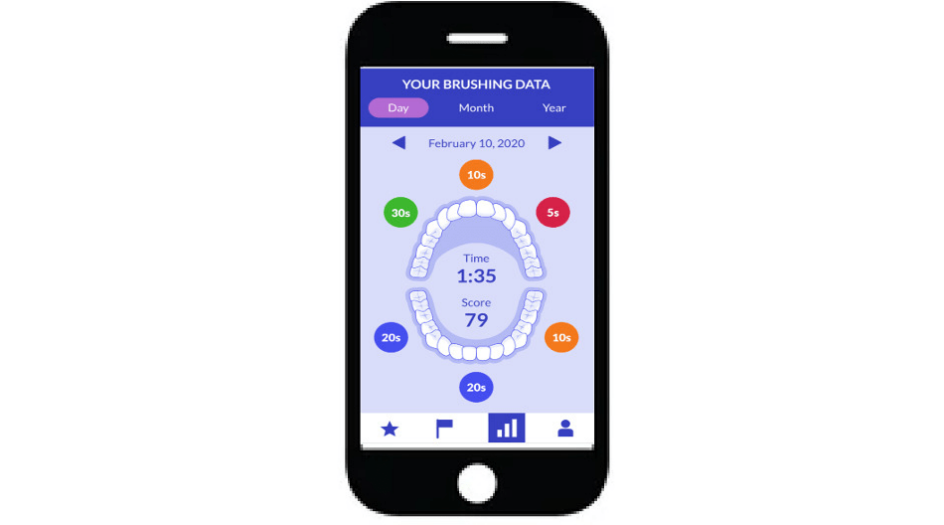
Oralytics interface.

By adopting a participatory design methodology and collaborating with a precursor group of 15 participants from community dental clinics, we created a supplementary smartphone app called Oralytics (Figure 1).

This app serves to give users feedback on their OSCBs. A distinct feature of Oralytics is its main interface, which offers session-based summaries detailing brushing activity and effectiveness. Using a recurrent probabilistic neural network algorithm developed by Oral B, the app translates sensor data into approximations of brushing coverage across different dental regions and also generates a composite score that reflects the thoroughness of each brushing session (see Figure 1). Additionally, Oralytics comes with a built-in text messaging function, paving the way for future delivery of educational content and tailored feedback at times chosen by the user. The app also includes analytical metrics to track user engagement, such as the frequency of accessing various app features and the total duration of interaction with the app.

### Study Setting

Participants were enrolled from November 2021 to July 2022 through UCLA-affiliated community dental clinics providing low-cost care to predominantly low-income individuals.

### Ethical Considerations

This study was classified as minimal-risk research and received approval from the UCLA institutional review board (IRB #18-000874). All participants provided written informed consent after undergoing a comprehensive informed consent process. This process included a detailed explanation of the study procedures, potential benefits and risks of participation, and the right to withdraw at any time. The informed consent document also clearly outlined how participant data would be anonymized and protected throughout the research.

To ensure participant confidentiality, brushing data were collected directly by the base charger and uploaded securely to a remote, central server. This server uses encryption to safeguard the data. Once uploaded, the data are aggregated and stored for future analysis. Additionally, all study and survey data were captured using UCLA's HIPAA-compliant REDCap (Research Electronic Data Capture) database, which offers further protection for sensitive information.

### Recruitment and Eligibility

Participants were sourced from the community dental clinics using a web-based platform designed for targeted stratified recruitment based on specific sociodemographic attributes. Built on the REDCap framework, the study management platform incorporated modules for eRecruitment, eScreening, eConsent, and eScheduling of virtual follow-up meetings with study staff [15-17]. Eligibility criteria included being an adult (18 years or older), owning a smartphone, being willing to use ROBAS for 4 months, and allowing their brushing sessions to be monitored remotely. Individuals who were edentulous or with physical or cognitive impairments that prevented them from using ROBAS were excluded. To reflect demographic diversity in the population, data were collected on gender, race (Black and non-Black), ethnicity (Latino and non-Latino), age group (18-29, 30-49, and 50+ years), and self-rated technological proficiency (low, moderate, and high). To maintain our intended sample size (N=40), we enrolled an additional participant for each previously enrolled participant who failed to complete the 4-month evaluation. Participants were compensated US $150 for their time and efforts and allowed to retain the Oral-B brush.

### Training and Onboarding

**Figure 2:**
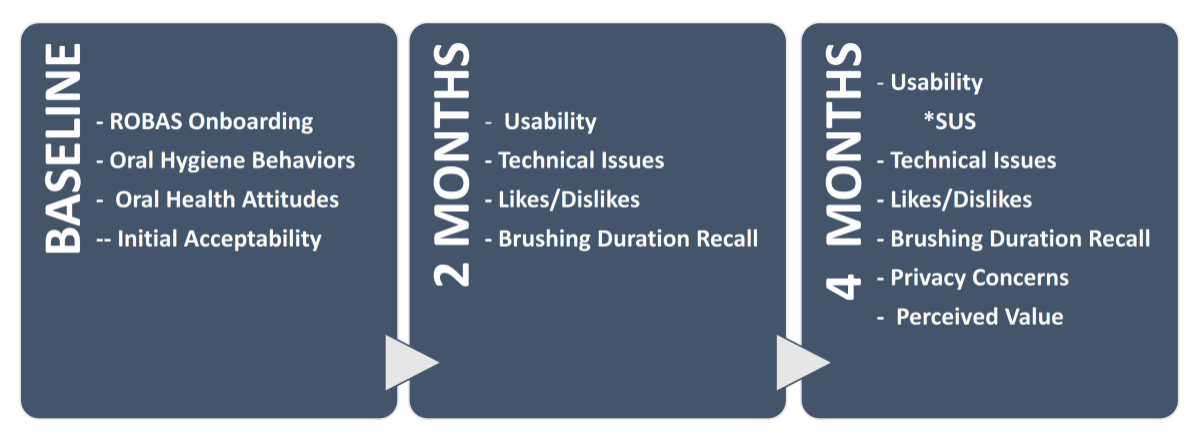
Study design with baseline, 2-month, and 4-month virtual surveys.

Figure 2 summarizes the study and the various surveys. Upon enrollment and consent, participants were mailed an Oral-B GENIUS X electric toothbrush along with detailed instructions, including an explainer video, for installing the Oralytics app and connecting to their Wi-Fi network. The setup and usage guidelines were accompanied by further instructions and onboarding, which were administered by research staff through a baseline virtual visit.

During an initial virtual meeting, research staff collected information on participants' typical toothbrushing routines, along with their knowledge, attitudes, and beliefs about oral health. Participants were directed to use ROBAS exclusively at home throughout the 4-month study period.

### Usability Measures

The System Usability Scale (SUS) was used as the primary instrument for assessing user acceptability and satisfaction. The SUS is a widely used, validated, and reliable tool for gauging the perceived usability of a system or product [18]. The SUS consists of 10 Likert-scale statements, with response options ranging from “Strongly disagree” to “Strongly agree.” Half of these statements are positively framed, with the other half negatively framed. Responses are scaled and summed to produce a total SUS score, which ranges from 0 to 100. Higher scores signify a more favorable user perception of the product overall. In conjunction with the total SUS score, we employed an adjective scale to offer greater insight into overall usability [18].

### Follow-Up Interviews

Research staff conducted a virtual follow-up survey 2 months into the study. This survey collects data on participants’ experiences with ROBAS, focusing on usability, acceptability, and any challenges they faced in adopting the system. They were also asked to recall recent brushing patterns over previous days.

After 4 months of use, the research staff conducted a virtual exit interview. The interview included both qualitative and quantitative components including SUS scoring, recall of recent brushing patterns, and privacy concerns. Additionally, participants were queried about how their use experiences influenced their perceived value of ROBAS, as well as their willingness to pay for it.

### Power and Sample Size Considerations

To ensure robustness in detecting usability issues, our study aimed for a final sample size of 40 participants. Faulkner [19] reported that an average of 99.6% of usability problems can be identified in formative testing with a sample size of 40, which also could be anticipated to yield estimates of correlations between ROBAS-recorded brushing time and self-reported brushing time within 0.15 of the true underlying correlation. With an expectation of 20%-30% attrition in study participation, we anticipated enrolling 50-58 individuals to obtain an analysis sample of 40. With this sample size, our study was powered to detect significant differences in effect sizes of 0.89 SDs with 80% power and 1.03 SD with 90% power across equal-sized subgroups.

### Statistical Analysis

Quantitative data were analyzed using R software system (version 4.2.3, R Foundation for Statistical Computing) [20]. *T* tests were employed to compare continuous variables like SUS scores across different subgroups of participants based on sociodemographic characteristics. Fisher exact tests were used for comparing categorical outcomes, such as the occurrence of technical issues, across subgroups. Correlations between self-reported and ROBAS-recorded brushing durations were explored using scatterplots.

Qualitative data, including in-depth interviews and open-ended survey responses, were anonymized and transcribed. Thematic analysis was conducted using ChatGPT to extract key quotes and identify overarching themes [21]. These themes were then categorized and quantified to allow for a more structured interpretation of the qualitative data. The qualitative data were then visualized using radar charts, also known as spider charts, to provide a comprehensive view of participants' perceptions of various dimensions of the ROBAS system [22].

To explore the relationships between baseline characteristics of participants and usability ratings, both univariate and multivariate linear regression models were used. These models integrated qualitative and quantitative data to provide a more holistic understanding of user experience and system feasibility.

## Results

### Sociodemographic Characteristics

The study cohort comprised 53 participants, consisting of 31 (58%) women and 22 (42%) men. In terms of race and ethnicity, 31 (58%) participants identified as Latino, while 22 (42%) identified as Black. Regarding age groups, 29 (55%) participants were in the 30- to 49-year age range, 14 (26%) were in the ≥50-year age group, and 10 (19%) fell into the 18- to 29-year age group.

Participants self-assessed their technology and smartphone proficiency, with the majority (n=31, 58%) considering themselves highly tech-savvy. Most others reported moderate tech-savviness (n=20, 38%), while a smaller number (n=2, 4%) reported low tech-savviness. Notably, none of the participants had previous experience using an electric toothbrush.

Out of the initial 53 participants, 40 (75%) completed the 4-month study including all associated surveys. There were no significant demographic differences between those who completed the study and those who withdrew (Table 1). Reasons for discontinuation, when provided, included constraints such as demanding work schedules and extended periods of travel.

**Table 1:** Demographic characteristics comparing participants who completed the study versus those who withdrew. *P* values were computed using chi-square tests (race and ethnicity) or Fisher exact tests (gender and age group). No significant difference in demographic breakdown between those who completed the study and those who dropped out was found for any group.

Variable		Number of participants				*P* value
		Completed the study (N=40), n (%)	Dropped out (n=13), n (%)			

**Gender**				.53		
	Female	23 (57.5)	9 (69.2)			
	Male	17 (42.5)	4 (30.8)			
**Race**				.58		
	African American	15 (37.5)	6 (46.2)			
	Not African American	25 (62.5)	7 (53.8)			
**Ethnicity**						
	Hispanic/Latino	24 (60.0)	8 (61.5)			
	Not Hispanic or Latino	16 (40.0)	5 (38.5)			
**Age group (years)**				.69		
	18-29	8 (20.0)	3 (23.1)			
	30-49	21 (52.5)	8 (61.5)			
	≥50	11 (27.5)	2 (15.4)			
**Tech savviness**				.82		
	High	25 (62.5)	7 (53.8)			
	Medium or low	15 (37.5)	6 (46.2)			

### Usability

**Figure 3:**
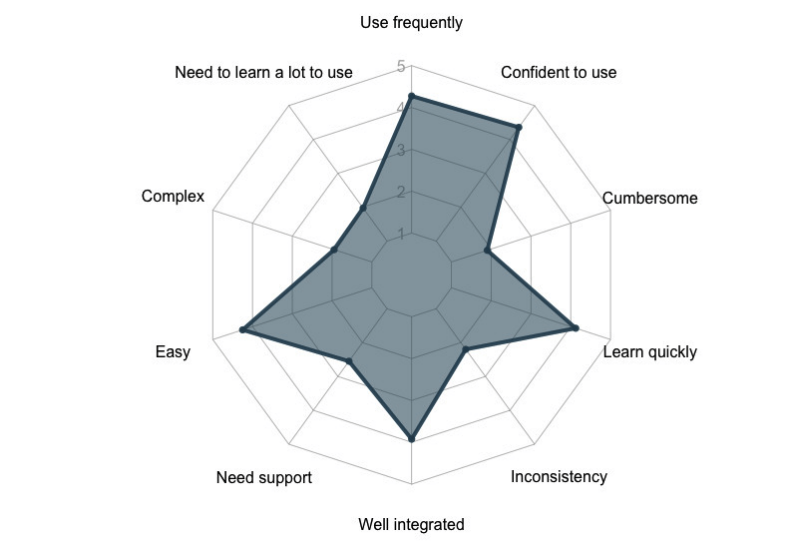
Radar chart demonstrating the average response to each of the 10 SUS survey questions. Higher scores represent higher degrees of concurrence with the respective sentiment.

In the baseline survey, 93% (n=37) of the participants expressed confidence in their ability to maintain oral hygiene using the ROBAS system and anticipated that the feedback from Oralytics would be valuable in enhancing their brushing habits. No significant difference was seen in expressions of the value of the ROBAS system across the levels of any participant's sociodemographic characteristics. Figure 3 captures the average response to each SUS survey question at the end of the ROBAS study.

**Figure 4:**
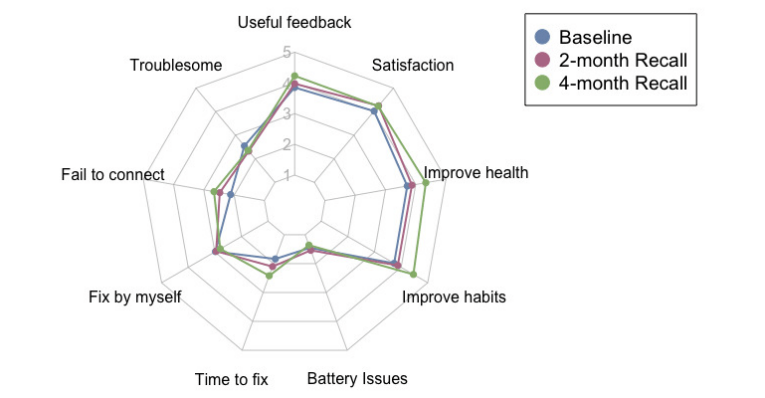
Radar chart demonstrating the average user sentiment toward ROBAS (Remote Oral Behaviors Assessment System) throughout the 4-month study. Higher scores represent higher degrees of concurrence with the respective sentiment.

Figure 4 encapsulates the participants’ impressions of various dimensions of ROBAS over the 4 months of use. Each radial of the spider chart signifies one of the items from the SUS. The opinions of the participants remained relatively consistent across the 3 surveys, with minor temporal variations observed in overall satisfaction levels and evaluations of the value of the ROBAS system.

Instances of technical issues decreased as the study progressed. However, when technical issues did arise, resolution times did not significantly decrease according to participant responses. Despite this, we did find improvements in the ability to handle common issues as the study progressed.

**Figure 5:**
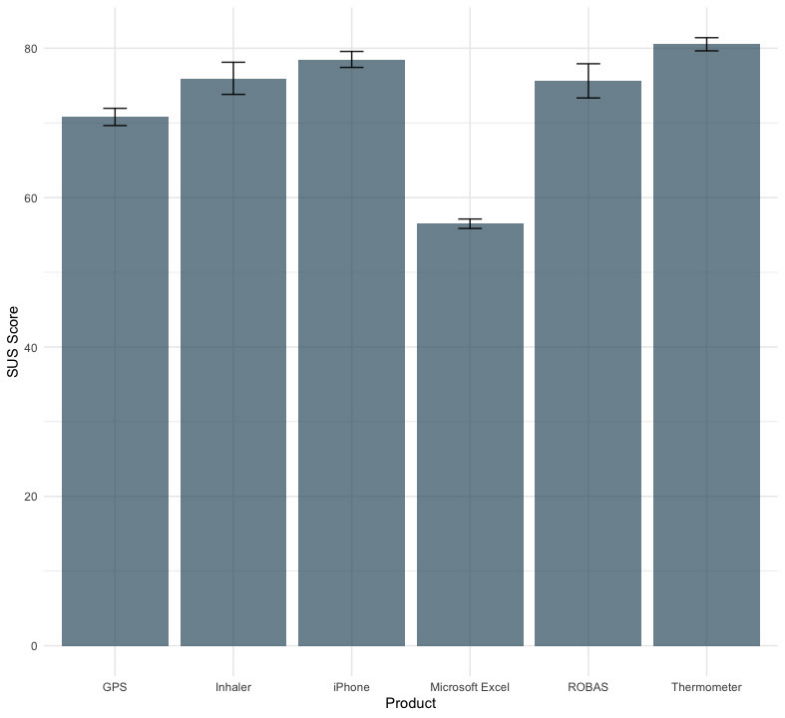
Average SUS scores of common medical and technological devices and services compared with ROBAS. ROBAS: Remote Oral Behaviors Assessment System.

SUS scores among those who completed the study had a mean of 75.6 (SD 14.5). Figure 5 consolidates SUS scores for ROBAS and compares them to previously reported SUS scores associated with various devices and technologies, including medical inhalers, iPhones, thermometers, GPS, and Microsoft Excel [23,24]. The average SUS score for the ROBAS system was within the range of values for other everyday devices and technologies. Also, the average SUS score did not differ significantly across the levels of any participant sociodemographic characteristics (Table 2).

**Figure 6:**
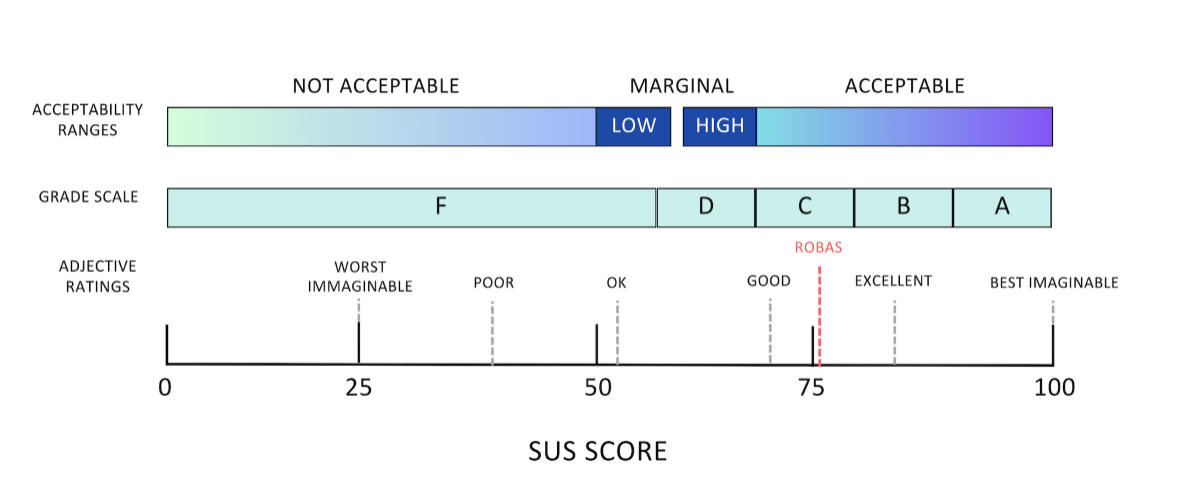
SUS (System Usability Scale) scores contextualized with different methods of rating overall usability, including acceptability ranges (not acceptable, marginal, and acceptable), grade scales (A, B, C, D, F), and adjective ratings (Worst imaginable, poor, ok, good, excellent, best imaginable).

When asked to evaluate ROBAS through an adjective-based scale, 28 (70%) respondents deemed the system excellent or better, 5 (12.5%) participants rated the system as good, and 7 (17.5%) participants considered the system to be “OK” (Figure 6). No participant labeled ROBAS as poor or worse. We saw no systematic pattern in the ratings across those levels of participant sociodemographic characteristics.

**Table 2:** System Usability Scale (SUS) scores across participant sociodemographic characteristics. SUS scores by group were compared using ANOVA and did not indicate any significant differences in the average SUS score within any sociodemographic group.

Variable			SUS score					*P* value
			Mean (SD)		Range			
			
Overall			75.6 (14.5)		35.0-100.0			
**Gender**								.29
	Female	77.6 (13.4)		52.5-100.0				
	Male	72.9 (15.8)		35.0-95.0				
**Race**								.58
	African American	77.3 (13.0)		52.5-95.0				
	Not African American	74.6 (15.4)		35.0-100.0				
**Ethnicity**								.29
	Hispanic or Latino	74.0 (15.4)		35.0-100.0				
	Not Hispanic or Latino	78.1 (13.0)		52.5-95.0				
**Age group (years)**								.74
	18-29	72.8 (16.6)		35.5-90.0				
	30-49	76.8 (13.4)		55.0-100.0				
	≥50	75.5 (15.9)		50.0-97.5				
**Tech savviness**								.54
	High	76.4 (12.0)		50.0-97.5				
	Medium or low	73.2 (17.6)		35.0-100				

### Feasibility

In terms of the feasibility of ROBAS in our target population, over the 4-month study period, 25 participants encountered a total of 28 technical challenges. The most prevalent issue, accounting for half of the reported problems (n=14, 50%), involved the smart charger inadvertently disconnecting or struggling to establish a connection with the home Wi-Fi. A smaller number of participants (n=4, 14%) reported occasional failures of the Oralytics app to launch, while others (n=5, 18%) noted instances where the app did not display their recent brushing data. The study team successfully resolved 86% of the reported issues (n=24) within an average time frame of 3.3 days. Importantly, there were no significant differences in the occurrence of technical challenges based on participant sociodemographic characteristics.

### Comparison of Self-Reported and Recorded Brushing Time

**Figure 7:**
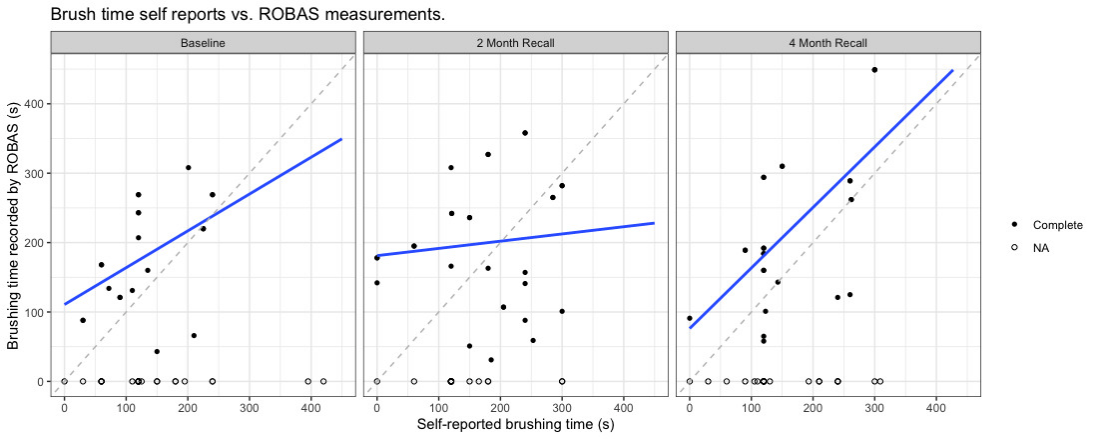
Correlation between recalled and ROBAS-measured brushing duration. ROBAS: Remote Oral Behaviors Assessment System.

During each survey session, participants were asked to provide an estimated duration of the time they spent brushing their teeth the previous day. This self-reported duration was then compared with the actual brushing time as captured by ROBAS. In the initial survey, there was a strong positive correlation (*r*=.80) between the self-reported and recorded brushing durations, indicating a high level of accuracy in participants' recollections. However, this correlation was markedly lower at the 2-month mark, dropping to a low *r*=.15, which highlighted a substantial discrepancy between participants' perceptions and the actual recorded times. By the end of the fourth month, the correlation improved to a moderate level (*r*=.62), suggesting that the accuracy of participants' self-reports had improved over the course of the study (Figure 7).

### Privacy Considerations

**Figure 8:**
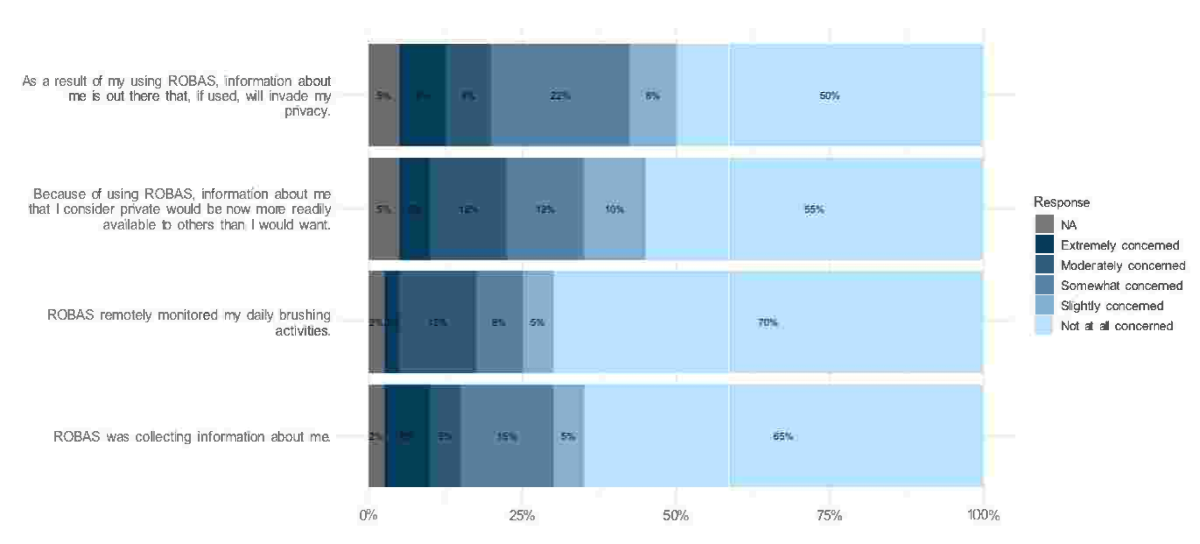
Levels of apprehension about privacy-related issues with the ROBAS. ROBAS: Remote Oral Behaviors Assessment System.

Figure 8 provides an overview of the participants' concerns related to privacy. For each question focused on privacy, a majority of participants—over 50%—indicated that they had no reservations about the privacy implications of using ROBAS, and overall, we observed that 50% of participants (n=20) expressed no reservations about privacy. Among the other 50% (n=20) expressing at least some concern about their privacy being invaded, we observed that 45% of participants (n=18) expressed concern about their private information being exposed, 30% (n=12) expressed concern that their brushing habits were being monitored, and 35% (n=14) had some level of concern about ROBAS collecting information about them. Across topics, a minimum of 1 out of every 8 participants voiced at least moderate concern for each question related to privacy. Our analysis did not reveal any significant correlation between these privacy concerns and the characteristics of the participants.

### Overall Impressions

The general sentiment toward ROBAS was largely favorable. A substantial portion of the participants (n=30, 75%) expressed a willingness to continue using a system akin to ROBAS for tracking their oral hygiene habits. Additionally, an overwhelming 87.5% (n=35) stated they would likely or very likely recommend ROBAS to friends and family. No specific subgroup of participants was identified as having notably higher or lower levels of approval.

Feedback from interviews with study staff at the baseline, 2-month, and 4-month time points offered diverse viewpoints on the system's strengths and weaknesses. Selected quotes from these interviews are available in Textbox 1. Features such as the timer, brushing habit tracking, pressure indicators, and the identification of neglected brushing areas received high praise. Users especially valued the grading system and daily feedback on their brushing performance.

**Key participant feedback.**

**Positive comments**

- “Love the brush; teeth feel and look a lot cleaner; the smart charger is great.”
- “I really like the system; the brush works really well.”
- “I notice a difference, especially in my gums and even with the tartar. I brush more frequently, and the brush keeps you accountable.”
- “I like the app; I am always wondering what I need to get a 100 score.”

**Negative comments**

- “At the beginning, I had issues getting the data to transfer from my brush; sometimes the toothbrush would disconnect causing me to need to reset the device so the data would work.”
- “The charger syncs well, but it gets dirty and disconnected easily. It’s more of a home base charger, so it’s difficult to travel with.”
- “Most of the time the brush wasn’t connected so the app wasn’t helpful.”

**Suggestions for improvement**

- “It would be helpful to have tips on how to brush better.”
- “The app could be more interactive – it felt flat.”
- “Notification on your phone for your brushing data – would encourage me to open the app more.”
- “A summary that you can export and send to dentists.”

On the flip side, criticisms were primarily aimed at issues with app connectivity, the cost of the system, and occasional technical hiccups. Users reported intermittent disruptions in the connection between the toothbrush and the app and found the troubleshooting process to be cumbersome. Some also mentioned that the app data were not always accurate or accessible.

Suggestions for enhancements mainly focused on improving the app's user interface and expanding the range of data displayed. Additional functionalities, such as tracking flossing activities and offering customization options, were also proposed. Other recommendations included providing a guide on different brushing intensities, reminders for brush head replacement, and a feature to immediately highlight missed areas during brushing

### Willingness to Pay

When asked about their readiness to invest in a system similar to ROBAS, participants indicated an average one-time payment willingness of US $60 (SD=US $22), with amounts ranging from US $18 to US $90. In terms of a subscription model, the average acceptable monthly fee was US $6 (SD=US $16). Overall, 97% (n=30) of those who responded to willingness-to-pay questions (n=31) expressed some willingness to pay for ROBAS, but, nearly 39% (n=12) of those who responded expressed reservations about committing to a monthly payment for a service such as ROBAS. No discernible trends were observed in the willingness to pay based on participant demographic characteristics.

## Discussion

### Principal Findings

Our investigation into the upgraded ROBAS system aimed to assess its ease of use and practicality for monitoring OSCBs in economically disadvantaged ethnic minority groups unfamiliar with electric toothbrush technology. The study revealed that ROBAS scored well in terms of usability, with an average SUS score of 75.6. This score is notably higher than the average SUS score of 68, as reported by Sauro [25] in 2011, and is competitive with usability scores for other technologies such as medical inhalers and smartphones [23,24].

Participants who were new to digital health platforms found ROBAS to be intuitive, easily incorporated into their daily lives and they expressed confidence in their ability to use the system. We found that of the 15 participants with low or medium self-rated digital savviness, 11 (73%) believed that the app and toothbrush were easy and enjoyable to use. These findings underscore the importance of user-friendliness in medical technologies, as ease of use is a critical determinant of user satisfaction and broader adoption [26].

Contrary to the stereotype that economically disadvantaged minority groups have low technological literacy [27], an overwhelming 96% of our study participants considered themselves to be moderately or highly tech-savvy. This aligns with other research indicating that minority groups, including African Americans and Hispanics, often rely on smartphones for internet access and are open to using for using mobile health (mHealth) technologies [28,29].

However, the study also identified challenges, primarily related to Wi-Fi connectivity. Inconsistent or poor-quality Wi-Fi emerged as a significant barrier, particularly for participants in low-income settings. Poor connectivity and Wi-Fi quality can be significant barriers to participation in low-income minority groups [29-33]. A smaller set of technical issues were related to occasional glitches in the Oralytics app. Our team was able to resolve 86% of these issues within an average of 3.3 days, highlighting the importance of robust technical support for such interventions.

Given that ROBAS relies heavily on stable home Wi-Fi connections, future versions should focus on mitigating these connectivity issues to offer a more consistent user experience. This is particularly crucial for low-income populations, who may face unreliable Wi-Fi connections, emphasizing the need for strong technical support systems when deploying mHealth technologies in these communities [34,35].

The varying relationship between recollection of recent brushing activity and actual measured values underscores the challenges affecting recall of self-care behaviors of low salience [9,36,37] while highlighting the potential for digital technologies to help individuals become more aware of their behaviors. At baseline, there was a strong positive correlation (*r*=.80), which dropped off considerably (*r*=.15) at the 2-month survey and increased to a moderate positive correlation (*r*=.62) at the 4-month exit interview. It is plausible that these fluctuations might be attributed to initial familiarity, habituation, and re-engagement. Due to the novelty of the electric brush, participants might initially be more attentive to their brushing activity and more accurate in their self-reporting. Over time, participants may become accustomed and may not monitor their brushing activity as closely. Toward the end of the study, participants had become more aware of their brushing patterns and better at estimating their activity levels. By making users more aware of their brushing behaviors, ROBAS has the potential to be integrated into digital behavior interventions that support individuals in staying engaged in optimal oral self-care.

Our results also indicate a willingness among economically disadvantaged ethnic minorities to adopt mHealth technologies for oral health management, even in the face of potential privacy concerns. This could imply that the level of concern about data privacy may be lower for toothbrushing activities compared to other contexts, or that the perceived benefits of using such a system outweigh the privacy risks. Our observations align with previous research indicating that the perceived benefits of digital health technologies may outweigh privacy concerns for some individuals [38-40]. Claudel et al (2020) [41] found that many individuals, including those from vulnerable populations, are willing to share personal health information with researchers and health care providers if they perceive that the benefits of using digital health technologies outweigh the potential privacy concerns. Although most participants reported no concerns about privacy issues related to ROBAS, a subset expressed some concerns about associated privacy issues. Other studies have reported privacy concerns as potential barriers to the adoption of mHealth technologies and highlight the importance of addressing privacy and security concerns in the design and implementation of digital health technologies, especially in vulnerable populations [40,42,43].

Although socioeconomically disadvantaged, most were willing to pay a one-time fee for a system like ROBAS and a large subset was open to a nominal monthly fee for the monitoring and feedback provided. Many indicated a desire to continue using ROBAS beyond the study and a substantial majority were willing to also recommend the system to their friends and families. While the exact amounts that individuals are willing to pay may vary depending on factors such as the perceived benefits of the system, their oral health values, and personal financial circumstances, our study shows that socioeconomically disadvantaged minorities see value in digital oral self-care systems and might be willing to pay for them if they believe the devices can help improve their oral health. This is in line with other studies reporting perceived value and comfort levels with digital health devices among individuals in under-resourced communities, particularly when these devices are seen as tools to improve access to health care services and promote better health outcomes [44,45]. Offering a range of payment options and pricing structures, as well as considering the unique needs and preferences of specific patient populations, could contribute to the uptake of mHealth systems like ROBAS.

Qualitative feedback from participants primarily centered on enhancing the user interface and expanding the range of data related to brushing activities. Participants also suggested the addition of features like flossing activity tracking, dental education, and customization options. Other recommendations included personalized coaching for improved brushing techniques, connectivity with dental care providers, and reminders for brush head replacements.

Our study had some limitations that limit generalizability. The study focused exclusively on urban, low-income minorities, so the results reported here might not extend to other populations. Subject drop-out can introduce bias, so variations in results could also have emerged from the roughly 25% of the initial participants who did not complete all study assessments. Furthermore, on account of modest sample sizes, the study did not have substantial statistical power to detect significant differences across participant subgroups, so it would not be surprising for such differences to emerge across larger sets of individuals.

### Conclusions

The research highlights ROBAS's promise as a digital platform for unobtrusively tracking daily oral self-care activities in marginalized communities. In the hands of low-income ethnic minorities with no previous electric toothbrush experience, ROBAS proved to be both feasible, evidenced by its stable and accurate data capture of brushing behaviors, and user-friendly, as reflected by strong SUS scores and positive user feedback. Influential factors for its uptake included ease of learning and operation, and feedback provided. These insights are critical for developing accessible, affordable, and effective mHealth solutions aimed at underserved communities.

## Abbreviations

DOSCI: digital oral self-care intervention
HIPAA: Health Insurance Portability and Accountability Act
mHealth: mobile health
OSCB: oral self-care behavior
REDCap: Research Electronic Data Capture
ROBAS: Remote Oral Behaviors Assessment System
SUS: System Usability Scale

## Data Availability

The data sets generated during and analyzed during this study are available from the corresponding author on reasonable request.
